# Diagnosis of a chronic spinal cord injury via televisit in a patient from an underserved community

**DOI:** 10.1093/jscr/rjab090

**Published:** 2021-04-19

**Authors:** Jeffrey M Breton, Keith M George, Ron I Riesenburger

**Affiliations:** Department of Neurosurgery, Tufts University School of Medicine, Boston, MA, USA; Department of Neurosurgery, Tufts University School of Medicine, Boston, MA, USA; Department of Neurosurgery, Tufts University School of Medicine, Boston, MA, USA; Department of Neurosurgery, Tufts Medical Center, Boston, MA, USA

## Abstract

A 42-year-old woman from a medically underserved community in rural New England was referred by her primary care provider (PCP) for televisit during the coronavirus disease 2019 (COVID-19) pandemic following 2 years of chronic neck pain and numbness in her left hand that was initially concerning for demyelinating disease. Upon further evaluation, it was revealed that she had experienced a traumatic fall with a concussion and symptoms consistent with central cord syndrome but had refused magnetic resonance imaging (MRI) at her initial medical evaluation. On MRI conducted 1 month prior to neurosurgical evaluation she was found to have a disc bulge and 4-mm T2-hyperintense lesion at the C4–C5 level that was consistent with a chronic spinal cord injury secondary to spinal trauma with associated vertebrogenic injury. This televisit confirmed the diagnosis of chronic spinal cord injury for this patient and allowed for discussion of future interventions, avoided further unnecessary referrals, and increased access to care.

## INTRODUCTION

The spread of coronavirus disease 2019 (COVID-19) has increased the use of telemedicine to connect physicians and patients in a “socially distanced” society. Telemedicine allows for virtual physician–patient interactions, whether on the telephone or using video conferencing software over the Internet. Even though frequently pictured as mainly a tool for primary care or chronic disease management, there too is a role for telemedicine in neurosurgery. In this case, a patient from an underserved community without access to a major academic medical center sought care via televisit to resolve an ongoing and diagnostically uncertain spinal cord injury without need for unnecessary further testing or referral.

## CASE DESCRIPTION

A 42-year-old female presented for neurosurgical evaluation with persistent neck pain and numbness in her left fingers 2 years after a traumatic fall on stairs with head strike without loss of consciousness. She initially presented to an outside hospital and on examination had left-sided neck and upper extremity (UE) pain, with paresthesias and reduced UE strength bilaterally and pain-limited lower extremity strength bilaterally. Her presentation was most concerning for a central cord syndrome spinal cord injury (SCI). Facial lacerations and bruising, headache and dizziness were concerning for a concussion. Brain and cervical computed tomography (CT) scans were negative for acute intracranial processes or cervical fractures, dislocations or spondylotic changes and she refused an MRI due to claustrophobia. Without MRI or flexion/extension radiographs, the outside hospital physician was not comfortable clearing her cervical spine. She was discharged maintaining a rigid cervical collar and recommended to follow up outpatient with neurosurgery.

By approximately 6 months post-injury she had regained most motor function and, without consulting a physician, self-discontinued the rigid cervical collar without any acute deterioration in neurological status. Her chronic neck and left shoulder pain and weakness did not improve with acetaminophen or occupational therapy. She did not disclose her fall history to her primary care provider (PCP) and was referred for neurosurgical evaluation. This occurred during a declared state of emergency in the northeastern USA due to COVID-19. The patient lived in rural New England, over 1 hour away from our hospital, and did not have the means to get to appointments, especially with public health measures in response to COVID-19.

**Figure 1:**
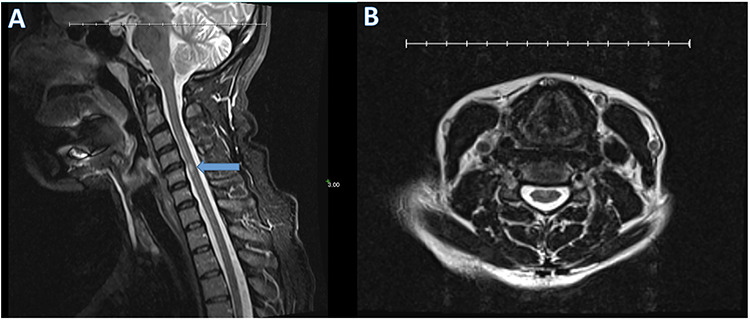
T2-weighted sagittal (**A**) and axial (**B**) cervical spine MRI reveals a 4 mm hyperintense area within the spinal cord at the C4–C5 level (arrow) and a disc bulge without spinal cord deformity.

During telemedicine evaluation, she discussed her fall. She had not understood the significance of her trauma and experienced symptoms consistent with postconcussive syndrome (PCS), including persistent problems with memory, concentration and decision making. Without telemedicine, she would have been advised to see a neurologist for brain and optic nerve MRI, visual evoked potentials, and lumbar puncture with cerebrospinal fluid analysis (CSF) to work up multiple sclerosis*.* A non-contrast T2-weighted cervical MRI from 1 month before neurosurgical evaluation revealed a 4 mm area of hyperintensity within the spinal cord at the C4–C5 level consistent with previous spinal cord injury (Fig. 1). Anteroposterior (AP), lateral and flexion/extension radiographs were recommended to assess for cervical instability, though these were not possible with COVID-19 restrictions. She was counseled regarding risks and benefits of future surgical decompression and fusion to prevent progression and stabilize the cervical spine, though surgery is unlikely to return function. After 1 year of relative clinical stability without her cervical collar, it was not deemed necessary to restart its application. Six weeks after evaluation she continued to experience neck pain with arm weakness and numbness without symptom progression.

## DISCUSSION

The use of telehealth has increased immensely since the advent of COVID-19 and, although likely to decline as practices resume in-person care options once more, many physicians have permanently adopted it. Past studies have examined the use of telemedicine to increase access in underserved communities, e.g. providing timely stroke care in Trinidad by allowing rapid assessment and designing care plans post-discharge [1]. Telemedicine also facilitates pre- and post-operative consultation between primary care providers and surgeons [2]. A randomized, controlled trial conducted in Spain revealed no difference in patient outcome or satisfaction in a General Surgery department for televisit versus in-person [3].

SCI is most common in the cervical region and approximately half have associated neurological deficits, with 85% of injuries to the cord during the initial trauma [4]. Care involves pre-hospital cervical immobilization, Advanced Trauma Life Support protocols, and the American Spinal Injury Association (ASIA) scoring system to evaluate for motor or sensory deficits [5].

Asymptomatic patients, defined by absence of midline cervical tenderness, absence of focal neurologic deficit, normal alertness and mentation, no intoxicants or confounders and no distracting injury, do not require imaging [5]. Otherwise, thin slice multidetector CT (MDCT) is preferred for initial evaluation following blunt trauma [4]. Cervical radiograph, with anteroposterior, lateral and open-mouth odontoid views, is an option when CT is unavailable [6]. MRI is preferred to assess for soft tissue injuries, i.e. to intervertebral discs, ligaments or the spinal cord. It is indicated in acutely injured patients with post-traumatic myelopathy, persistent neurologic deficits or when clinical findings do not align with radiograph/CT findings and practitioners cannot complete a full neurologic exam. Maintaining rigid cervical collars may be indicated until a patient is asymptomatic, has normal flexion/extension radiographs with adequate visualization, has a normal MRI taken within 48 h of injury or at the discretion of the treating physician [6].

Postconcussive syndrome is considered in patients experiencing continued concussion symptoms, such as headache, poor concentration, dizziness, imbalance, depression, anxiety or stress intolerance that persist beyond the predicted timeframe for resolution of a mild traumatic brain injury [7]. In this patient’s case, it is possible that the concussion and likely PCS limited her decision making and understanding such that she refused initial MRI evaluation and outpatient follow-up.

SCI care has already benefitted from the use of telerehabilitation after hospital discharge [8] and in treatment and follow-up for SCI [9]. This case demonstrates the convergence of a previously undiagnosed and undertreated chronic SCI with telemedicine, illustrating how televisits address health care access disparities that have been exacerbated by a global pandemic. To the best of our knowledge, this is the first report of telehealth used to confirm a chronic spinal cord injury following a missed initial diagnosis.

## CONFLICT OF INTEREST STATEMENT

None declared.
